# Conceptualising and operationalising resilience in older adults

**DOI:** 10.1080/21642850.2019.1593845

**Published:** 2019-03-28

**Authors:** Theodore D Cosco, Almar Kok, Andrew Wister, Kenneth Howse

**Affiliations:** a Gerontology Research Center, Simon Fraser University, Vancouver, Canada; b Oxford Institute of Population Ageing, Oxford University, Oxford, UK; c Department of Epidemiology & Biostatistics, VU University Medical Center, Amsterdam, the Netherlands

**Keywords:** Resilience, conceptualisation, operationalisation, older adults, methods

## Abstract

**Context:** As a result of increases in life expectancy and decreases in fertility, the proportion of the population entering later life has increased dramatically in recent decades. When faced with age-related challenges, some older adults respond more positively to adversity than would be expected given the level of adversity that they have experienced, demonstrating ‘resilience’.

**Objectives:** Having a clear conceptual framework for resilience is a prerequisite to operationalising resilience in a research context.

**Methods:** Here we compare and contrast several approaches to the operationalisation of resilience: psychometric-driven and data-driven (variable-centred and individual-centred) methods.

**Results:** Psychometric-driven methods involve the administration of established questionnaires aimed at quantifying resilience. Data-driven techniques use statistical procedures to examine and/or operationalise resilience and can be broadly categorised into variable-centred methods, i.e. interaction and residuals, and individual-centred methods, i.e. categorical and latent class.

**Conclusions:** The specific question(s) driving the research and the nature of the variables a researcher intends to use in their adversity-outcome dyad will largely dictate which methods are more (or less) appropriate in that circumstance. A measured approach to the ways in which resilience is investigated is warranted in order to facilitate the most useful application of this burgeoning field of research.

As a result of increases in life expectancy and decreases in fertility, the proportion of the population entering later life has increased dramatically in recent decades (United Nations, 2010). Advances in public health, health care, and medical technology, have realised unprecedented extensions in the length of life. With more individuals living longer, there has been an increase in the proportion of the population facing age-related disorders and disease; age has been widely established as one of the strongest predictors of acquiring multiple morbidities (Wister et al., 2016). Consequently, in addition to research into increasing the quantity of life, there has been mounting interest in addressing the quality of life in these additional years, particularly within the context of age-related functional limitations and disease.

When faced with age-related challenges, some older adults fare better than others. Individuals that respond more positively to adversity than would be expected, given the level of adversity that they have experienced are described as demonstrating ‘resilience’ (Windle, 2011). The field of resilience research has its roots in developmental psychology; the first resilience studies were conducted with children growing up in adverse environments (Garmezy, Masten, & Tellegen, 1984; Rutter, 1987). Children that were able to navigate these challenges and avoid pathologies in later life were described as being ‘resilient’ (Garmezy et al., 1984; Rutter, 1987). Since these foundational studies of resilience in children, the literature has expanded from investigations in early life to examining resilience in mid- and later-life, and among a variety of vulnerable groups, from the perspective of many disciplines, e.g. epidemiology, gerontology, and sociology (Ong, Bergeman, & Boker, 2009).

Although these expansions have illuminated resilience from different perspectives and highlighted many ways in which resilience manifests in individuals, a consensus definition and operationalisation has yet to emerge (Cosco, Kaushal, et al., 2017). This lack of universal operationalisation stems from the diversity of the constituent components of resilience and the operational frameworks used to define resilience. Having a clear conceptual framework for resilience is a prerequisite to operationalising resilience and subsequently identifying variables that are associated with resilience (Cosco, Howse, & Brayne, 2017; Cosco, Wister, Brayne, & Howse, 2018). For researchers developing primary data collection studies and those using secondary data, having a firm grasp on the ways in which resilience can be operationalised is an asset. Within a life course perspective a number of methods have been employed to capture resilience cross-sectionally and longitudinally (Cosco, Kaushal, et al., 2017). It is important to note, that the ways in which resilience manifests itself may change across the life course and for different types of adversity (e.g. loss of spouse, residential move, environmental catastrophe, or multimorbidity); the challenges faced by individuals aged five versus 95 are vastly disparate. Consequently, the ways in which individuals demonstrate resilience may vary across the life course, requiring careful examination and conceptual framing in order to support evidence-based operationalisation methods. Previously, Miller-Lewis, Searle, Sawyer, Baghurst, and Hedley (2013) have examined cross-sectional methods for operationalising resilience in children; here, we expand upon this work through a contextualisation of resilience operationalisations within older adult populations, the inclusion and comparison of longitudinal methods, with recommendations for future applications of resilience methods in older adult research.

## Operationalising resilience

There are several approaches to the operationalisation of resilience: psychometric-driven and data-driven (variable-centred and individual-centred) methods. In this section these methods will be outlined within the context of research on older adults and the relative strengths and limitations compared. This information can then be used to inform the development of prospect resilience projects, through the assessment of the relative suitability of operationalisation methods within the context of a given study.

### Psychometric-driven methods

Psychometric-driven methods involve the administration of established questionnaires aimed at quantifying resilience, e.g. Wagnild & Young’s Resilience Scale (2003), the Brief Resilience Scale (Smith et al., 2008), and the Connor-Davidson Resilience Scale (2003). This type of study requires that a resilience scale is included, a priori, in the battery of instruments implemented in primary research. To date, a study with a resilience scale as the primary focus of a longitudinal analysis has not been conducted (Cosco, Kaushal, et al., 2017). Consequently, psychometric-driven resilience studies are best suited to prospective projects rather than implementing these methods in secondary data analyses.

A recent systematic review of resilience scales conducted in older adult samples revealed only six studies that had been conducted in which psychometric analyses were performed (Cosco, Kaushal, Richards, Kuh, & Stafford, 2016). In order for a researcher to be confident that the scale they are employing is capturing the intended phenomenon in a sample that differs from the sample in which the scale was developed, validation studies must be conducted. The majority of resilience scales have been developed in younger populations; therefore, validations of these scales in older adults are necessary to confirm that resilience is being captured in the same way. Given the diversity of adverse events occurring across the life course, investigations into whether resilience will manifest in the same ways and will be captured in the same way via resilience scales must be conducted. A recent systematic review provides some supporting evidence for the psychometric robustness of the three scales captured in the review, i.e. The Resilience Scale (Wagnild, 2003), the Connor Davidsonscale (Connor & Davidson, 2003) and the Brief Resilient Coping Scale (Sinclair & Wallston, 2004); however, none of the psychometric evaluations of resilience scales in older adults conducted to date are properly comprehensive, for example: there is no consensus as to the dimensionality of these scales’ latent structure.

In order to assess the psychometric robustness of a resilience scale a number of reliability and validity analyses must be conducted. All of the studies captured in the resilience scale psychometric properties review used Cronbach's alpha, an indicator of the degree to which components on a scale are all measuring the same construct (Cosco et al., 2016), to assess the scale's internal reliability. Several studies in the review used Cronbach's alpha alone, as a test of psychometric robustness (Cosco et al., 2016). Whilst the values identified in the analyses are encouraging, i.e. all >.80, these results would benefit from further psychometric analyses; internal consistency is only one of a suite of psychometric properties necessary to conclusively verify the robustness of a scale (Dima, 2018; Watson, Egberink, Kirke, Tendeiro, & Doyle, 2018).

Several studies of resilience scales in older adults have augmented their internal consistency analysis with convergent validity, i.e. positive correlation with scales capturing similar constructs, and discriminant validity, i.e. inverse correlation with scales measuring divergent constructs, measures and factor analysis. When compared to depression scales, e.g. Beck Depression Inventory (Beck, Ward, Mendelson, Mock, & Erbaugh, 1961), significant negative correlations are observed with resilience (Cosco et al., 2016). Further, significant positive correlations with resilience scales in older adults have been observed with scales of general health, self-mastery and social support (Bousquet et al., 2015). Whilst these correlations have strong face validity, given the indirectly observable and presumably dynamic nature of resilience, it is hard to know what sorts of correlations should be observed for these constructs as they relate to resilience.

An examination of the factor structure of resilience scales used in older adults demonstrated divergence from the originally proposed, i.e. established, structures (Cosco et al., 2016). These results may indicate that resilience scales are not capturing the same phenomena in younger and older adults or this could be explained by methodological artefacts and questionable methods of factor extraction. In studies conducting factor analysis with a number of age groups, ethnicities and geographic locations, the original factor structure for resilience scales, e.g. Connor Davidson (Masten & Oconnor, 1989), have not been replicated, e.g. American college students (Campbell-Sills & Stein, 2007), military veterans (Green et al., 2014), Chinese adults (Yu, Lau, Mak, Zhang, & Lui, 2011), and Australian adolescents/ adults (Gucciardi, Jackson, Coulter, & Mallett, 2011). All of the studies conducting exploratory factor analysis either did not report their means of extraction (Lamond et al., 2008) or have used the Kaiser criterion. The Kaiser criterion is suggests extraction factors based on Eigenvalues >1, as the sole means of factor extraction, has been almost universally viewed as ineffective (Steger, 2006).

### Strengths and limitations

When resilience scales are employed in a study, the values can be used in a variety of circumstances, e.g. as a determinant, outcome, covariate, mediator, etc. As a result of being a standalone variable that does not require other variables to be incorporated into its calculation, the values of a resilience scale are analytically versatile. Researchers can then use these values as they see fit within their studies.

Resilience scales must be included in the initial survey administration of a research project in order to be analysed. This is a considerable drawback for the study of aging, where longitudinal methods are commonly used and data collected decades prior to analysis. Consequently, unless a resilience scale has been included in the original study, these data cannot be analysed; a considerable obstacle for researchers using secondary datasets.

An underlying assumption of resilience scales is that resilience is uniformly manifested across the life course. To date, a resilience scale has not been developed specifically with older adults in mind. In choosing a resilience scale to employ in a prospective study, researchers must be cognisant of the properties of that scale, its applicability in older adult populations, and whether or not the scale is capturing the form of resilience the researchers intends to capture.

Although the existing evidence of the psychometric robustness of resilience scales in older adults has been positive, there is substantial room for further investigation using expanded psychometric analysis, e.g. latent structure, and improved statistical techniques.

### Data-driven techniques

Data-driven techniques use statistical procedures to examine and/or operationalise resilience. These methods can be broadly categorised into variable-centred methods, i.e. interaction and residuals, and individual-centred methods, categorical and latent class.

## Variable-centred methods

### Interaction

Using statistical interaction effects, it is possible to identify under which circumstances the effect of adversity on a given outcome is reduced. For example, physical limitations can be considered a typical risk factor for decreased wellbeing. However, one might hypothesise that having more socioeconomic resources may serve as a protective factor that reduces this effect. Statistical interaction identifies variables that protect against negative outcomes particularly in a context of adversity, buffering adverse events and contributing to higher levels of positive adaptation, i.e. ‘resilience’ or ‘protective’ factors. To test the hypothesis that socioeconomic resources are a protective factor, buffering the effects of physical limitations on wellbeing, a regression model is employed to examine the association between adversity, i.e. physical limitations, and outcome variables, i.e. wellbeing, with an interaction term, i.e. socioeconomic position (SEP), included in the model of the level of wellbeing associated with physical limitations is weaker at a high SEP than at a low SEP, this would suggest that SEP could be considered a resilience factor. Variables demonstrating that the adversity-outcome relationship is weakened at higher levels, can then be identified as potential targets for interventions as being ‘protective’ factors.

### Strengths and limitations

Through the identification of synergistic (or antagonistic) variables in the outcome-adversity relationship, it is possible to identify potential targets for intervention. Resources identified as potential targets can then be investigated further using experimental methods and interventions tailored to foster greater resilience in older adults.

In the use of the interaction method, however, resilience is never directly quantified, which presents a significant limitation to this method. In contrast to psychometric-driven methods, in which a single value is used to quantify resilience, statistical interaction never quantifies resilience; therefore, it can only be applied to identify resources that may increase resilience in some individuals. Further, whether a resource is protective is dependent on the strength of the effect of a potential protective factor in individuals without adversity. If the effect is equally strong in those without adversity, no interaction effect will be found; however, this does not mean that the factor is not protective against adversity, but only that it is not more protective than in those without adversity. Consequently, the criteria for identifying ‘true’ resilience factors via interactions are quite stringent. It also must be taken into consideration that establishing causality is difficult in observational studies, if not impossible in cross-sectional studies, limiting the scope of applicability of these methods. Further, these relationships are often not identified due to the high statistical power, i.e. large sample sizes, required and the inability to simultaneously model multiple (synergistic) protective factors.

### Residuals

**Figure 1.**
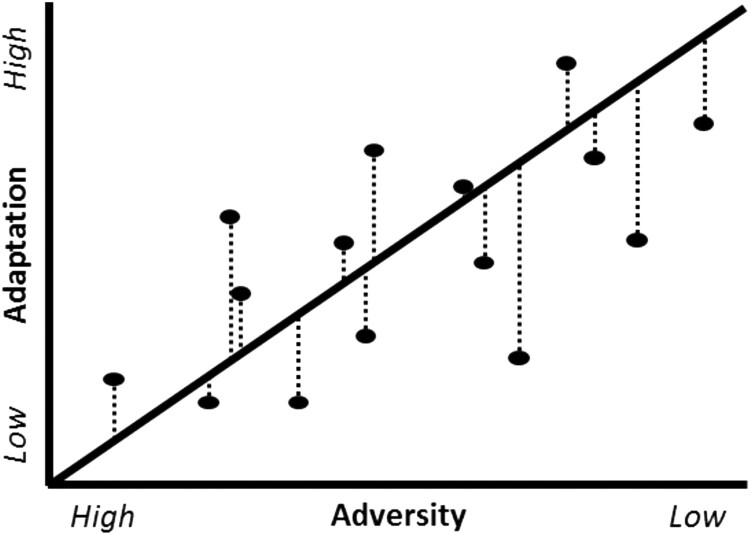
Plot of adaptation linearly regressed on adversity in a continuous residuals model. * The residual values, i.e. distance from the fitted regression line indicated by the dotted line, quantify the level of resilience.

The residuals procedure plots a regression between scores capturing adversity and scores capturing positive adaptation to that adversity, e.g. having a high quality of life or absence of chronic pain despite adversity. The raw residual values can be used as a continuous measure of resilience or the values can be categorised based on an a priori threshold to identify individuals as resilient. The continuous residuals method allows for the full utilisation of the sample providing greater power than categorisation methods and provides a level of granularity that is not possible with categorisation methods. However, in order to make use of this method the data used in the adversity/adaptions must be continuous and conform to the assumptions necessary for linear regression. Non-normal data can be transformed, but issues such as multicollinearity and heteroscedacity are more difficult to address. This method has previously been used to quantify cognitive reserve (Zahodne et al., 2015) as well as resilience (Cosco, Cooper, Kuh, & Stafford, 2018) (Figure 1).

**Figure 2.**
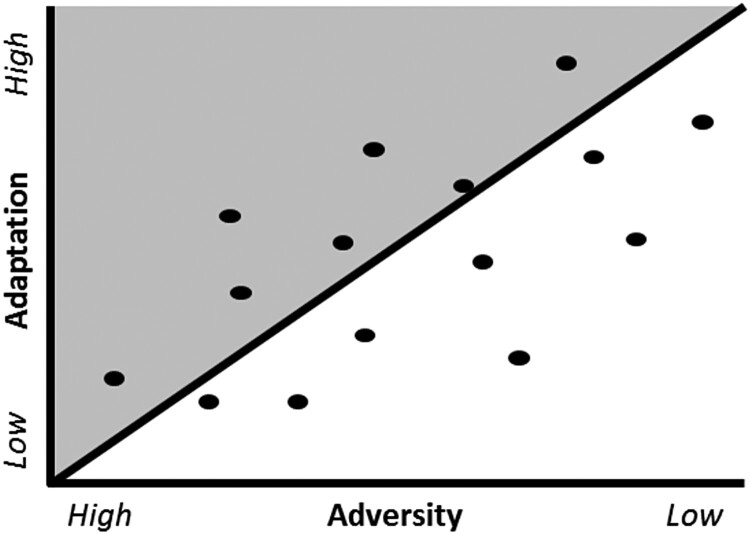
Plot of adaptation linearly regressed on adversity in a categorical residuals model. * The shaded area identifies individuals as ‘resilient’.

Categorical residual methods may use a combination of definition-driven and data-driven methods. The residuals to be categorised utilise the data-driven techniques; however, the means with which these data are classified are definition driven. There are several ways in which individuals may choose to divide residuals in the categorical method, such as having everyone above the fitted line or the top tertile is defined as ‘resilient’. In dividing the original sample into individuals that are resilient or not, there is some loss of power, which may obscure significant relationships in small samples. Further, division of the sample into smaller groups may also lead to the identification of spurious relationships (Figure 2).

### Strengths & limitations

Through the use of regression residual values, we can quantify resilience on a continuum of resilience to vulnerability. These data are continuous, normally distributed, and maximise the sample's statistical power, which allows for a variety of analytical procedures to be performed. This versatility in analysis allows for more granular output and greater insights into the relationship between the exposure and resilience.

Both the outcome and adversity variables must be suitable to be fitted to a regression model. Therefore, the nature of the variables to be examined and the relationship between these variables must be investigated and meet regression assumptions before this method can be applied. Consequently, it may not be possible to conduct these analyses based on the nature of the variables included in the model, limiting the scope for application of these methods.

The continuous residual model provides a more granular, continuous quantification of resilience, whist the categorical residual method dichotomises this continuum. Individuals with positive residual values are deemed to be resilient and those with negative values are not resilient. This method makes the interpretation of analysis much easier, but at the expense of granularity. The nuance captured in the relative levels of resilience is lost, which may obscure aspects of the model, e.g. outliers.

## Individual-centred methods

In contrast to variable-centred methods that focus on associations between variables, individual-centred methods focus on defining subgroups of individuals with particular combinations of features. In the Researcher-driven thresholds approach, individuals’ level of adversity experienced and their response to that adversity is used and in the latent class approach a combination of adversity and pattern of functioning across multiple time points is used.

### Researcher-driven thresholds

Individual-centred methods that use researcher-driven thresholds utilise scores that individuals attain on adversity and positive outcome variables to identify individuals as resilient, based on cut-offs set by the researchers. Generally, these methods are established by researchers a priori, such as in Masten and Obradović (2006) (Table 1). In Masten's model, the researchers chose, a priori, to divide the outcome and adversity variables into tertiles in a three-by-three matrix. Individuals that experience the greatest adversity, i.e. top adversity tertile, but also demonstrate the best outcomes, i.e. top adaptation tertile, are considered ‘resilient’. This model takes into account the variety of adversity-outcome relationships by categorising individuals into additional categories beyond ‘not resilient’, i.e. maladaptive, highly vulnerable, and competent. Subsequently, factors related to resilience are determined by drawing an extensive profile of, for example, psychological and social characteristics of the individuals in each group, and statistically testing differences in these profiles between the resilient group and the other groups. Characteristics in which the resilient differ favourably from the maladaptive group qualify as protective factors, and characteristics in which the resilient also differ favourably from groups of individuals who were not exposed to adversity would provide even stronger evidence for the protective factors.

**Table 1.** Masten's resilience categorisation matrix.

		**Adaptation**		
		Lowest Tertile	Middle Tertile	Highest Tertile
**Adversity**	Highest Tertile	Maladaptive	X	***Resilient***
	Middle Tertile	X	X	X
	Lowest Tertile	Highly Vulnerable	X	Competent

### Strengths & limitations

This form of a priori threshold usage is advantageous in its capacity to accommodate a range of variable types. In contrast to the residual modelling procedure that has a number of assumptions that must be met with respect to the relationship between variables, this categorisation procedure can accommodate ordinal and continuous variables. Further, the identification of a number of groups with unique adversity-outcome relationships permits more nuanced examination than a binary resilient vs not resilient analysis, e.g. as per the categorical residual method. This method most closely aligns with the resilience conceptual framework of ‘functioning better than expected given the level of adversity experience’ – as it focuses on individuals who have demonstrably attained good outcomes despite adversity. Furthermore, analyses, e.g. ANCOVA, provide a more direct insight into the characteristics associated with resilient individuals.

In the categorisation of individuals into a variety of adversity-outcome groups, statistical power is diminished. The ‘resilient’ group identified via this method will generally be a small sub-sample of the study participants, which may compromise the analysis. Particularly in small samples, maintaining as much power as possible is an important analytical strategy; therefore, this method may not be appropriate in circumstances in which relationships may be obscured via under-powered analyses. Further, the thresholds at which individuals are identified as ‘resilient’ are established a priori, based on researchers’ decisions. Research into identifying thresholds of resilience in which interventions are particularly effective has not been conducted to date. This research could be used to inform the establishing of a priori categorical thresholds.

### Latent class approach

Another individual-centred approach to operationalise resilience uses data-driven techniques to detect different types of responses to adversity in a particular study sample, is latent class analysis. These techniques divide individuals into subgroups on the basis of a predefined set of variables, observed once or repeatedly. In the study of resilience, these methods are often applied to longitudinal datasets, and provide a typology of trajectories in a given outcome (e.g. depressive symptoms) following a significant stressor (e.g. bereavement) (examples of studies adopting this approach are (Bonanno & Mancini, 2012; deRoon-Cassini, Mancini, Rusch, & Bonanno, 2010; Galatzer-Levy & Bonanno, 2012)). As such, these methods capture heterogeneity in individual responses to adversity, of which one or more detected trajectories may be evaluated as ‘resilient’. Subsequently, characteristics of resilient individuals can be compared with individuals with other trajectories, resulting in the identification factors that are associated with resilience.

The most common techniques applied within this approach are latent class models. Based on mathematical algorithms, these techniques are used to search for a division of a sample into subgroups that maximises differences in outcomes between subgroups while minimising differences within subgroups. The number of subgroups that provides the optimal statistical fit to the data is not automatically retrieved by these algorithms, but is determined by an iterative process. First, a model with two classes is fitted, after which a number of statistical indicators is evaluated by the researcher. Then, a model with three classes is fitted, and the fit of this model is compared to the model with two classes. This process is repeated with three, four, or n classes until the optimally fitting number of classes is found (Nylund, Asparouhov, & Muthén, 2007). In addition to statistical indicators, it is recommended that theory and substantial interpretation of the identified types of trajectories guide model selection (Muthen, 2003). Most previous studies of resilience have used Growth Mixture Modelling (GMM; (Muthén, 2006)) or Latent Class Growth Analysis (LCGA; (Nagin, 1999)), which are technically similar. In LCGA, the model assumes that there is no within-class variance in trajectories. That is, everyone within a latent class is assumed to have exactly the same intercept and slope parameters. In GMM, within-class variance is allowed. Because of the constraints on the model, LCGA is computationally less demanding and less often results in model estimation problems, but tends to overestimate the number of latent classes (Tofighi & Enders, 2008).

After the optimally fitting model is decided upon, categorical variables expressing each individual's most likely latent class membership can be used in subsequent analyses to determine factors associated with resilience (e.g. using multinomial logistic regression models in which latent class membership is the dependent variable).

### Strengths and limitations

Latent class models are powerful tools for researchers to get a grasp on the heterogeneity in functioning before and after adversities that often occur in ageing populations, e.g. hospital admission, functional decline, and bereavement. Compared to other methods of operationalising resilience, this approach is a good choice for examining longitudinal data. Furthermore, the division of samples into subgroups is based on the variation present in the study sample, and may therefore be more objective than researcher-defined divisions. Additionally, LCGA and GMM are very flexible methods. For example, they can easily accommodate complex growth parameters (e.g. quadratic and cubic slopes), model multiple parallel outcome trajectories, or estimate separate pre- and post-adversity trajectories (i.e. ‘piecewise’ models); (Kim & Kim, 2012).

Limitations are that it is not known in advance whether a group that can be evaluated as being resilient will emerge from the analysis and will be large enough to allow for properly powered statistical tests. Furthermore, several aspects of LCGA and GMM are under debate, for instance whether the hypothesised subpopulations reflect ‘real’ subpopulations, how one should decide on the optimal number of classes, how one should deal with the potential impact of the choice of growth parameters on the results, whether or not to constrain within-group variance, and what to do if statistical indicators of model fit contradict each other (Bauer & Curran, 2003; Infurna & Grimm, 2017; Ram & Grimm, 2009). Finally, because the results are based on the variation present in a specific dataset, they may have low comparability to identical analyses in other datasets.

### Recommendations & future directions

The diversity of methods for conceptualising and operationalising resilience presents both challenges and opportunities. In the absence of a consensus definition it is difficult to make cross-study comparisons and the language surrounding the resilience literature may become somewhat obscure. Further, given the interchangeability of the components of resilience, particularly with regards to adversity-outcome dyads, it is feasible that a variable could be an adversity in one instance and an outcome in another instance, depending on the temporal nature of the variables. For example, psychological distress in mid-life could be an adversity variable and physical disability in later life an outcome, or vice versa: physical disability in mid-life could be an adversity whilst psychological distress in later life could be the outcome. Therefore, it is difficult to generalise about the factors that contribute to resilience across a variety of adversity-outcome dyads.

The specific question(s) driving the research and the nature of the variables a researcher intends to use in their adversity-outcome dyad will largely dictate which methods are more (or less) appropriate in that circumstance. As noted above and summarised below in Table 2, each method has its strengths, limitations, and underlying assumptions that must be taken into consideration before being used. Broadly, one is advised to choose an approach that maximises granularity and statistical power whilst also producing an outcome that best addresses the hypothesis being tested.

**Table 2.** Methods of operationalising resilience and their strengths and limitations.

		Variable-centered			Individual-centered	
	Psychometric-driven	Interaction	Residuals		Researcher-driven Thresholds	Latent Class Approach
			Continuous	Categorical		
Acceptable Variable Types	n/a	Ordinal, continuous	Continuous	Continuous	Nominal, ordinal, continuous	Nominal, ordinal, continuous
Minimum Sample Size Requirements	No	Yes	Yes	Yes	No	Yes
Statistical Power	n/a	Low	High	Low	Low	Depends on sample
Granularity^a^	Moderate	Depends on measurement variables	High	Low	Low	Variable
Resilience Quantified	Yes	No	Yes	Yes	Yes	Yes
Limitations	Must be included in existing dataset or collected prospectively	Individuals never explicitly identified as ‘resilient’	Restrictive criteria for included variables	Restrictive criteria for included variables	Subjective group membership criteria, i.e. set by researchers	Group sizes are not known in advance
Strengths	Integrate levels of resilience into a single psychometrically-robust score	Identifies targets for intervention	Greater insight into adversity-outcome relationship	Interpretation of analysis very straightforward	Accommodates a range of variable types	Effective method for longitudinal studies

^a^Level of detail possible.

In the context of applying diverse methodological approaches to resilience, it is unlikely that relationships exist strictly as methodological artefacts. If the same resources are identified as fostering greater levels of resilience in individuals regardless of the method used, this provides more robust evidence for this relationship. Conversely, if a single method is used, e.g. a resilience scale, it could be that there is a component specific to that scale driving an association with a protective factor rather than the underlying construct of resilience itself.

As the population progressively shifts towards a greater population of older adults, researchers, clinicians and health providers will need to find ways to foster more positive responses to age-related challenges. Fostering better outcomes via resilience is a potential avenue with which to pursue this end goal. There are myriad ways to measure and operationalise resilience, with a few of these methods highlighted above. Depending on the nature of the variables in the study, e.g. categorical or continuous, and the intended output from the study, e.g. identification of resilience recourses, some methods may be more or less appropriate for these specific applications. Consequently, a measured approach to the ways in which resilience is investigated is warranted in order to facilitate the most useful application of this burgeoning field of research.
